# Multicenter retro-prospective observational study on chronic hypoparathyroidism and rhPTH (1–84) treatment

**DOI:** 10.1007/s40618-022-01800-y

**Published:** 2022-04-23

**Authors:** G. Marcucci, G. Beccuti, G. Carosi, F. Cetani, L. Cianferotti, A. M. Colao, C. Di Somma, M. Duradoni, A. Elefante, L. Ghizzoni, M. Giusti, A. G. Lania, E. Lavezzi, B. Madeo, G. Mantovani, C. Marcocci, L. Masi, S. Parri, F. Pigliaru, A. Santonati, A. Spada, L. Vera, M. L. Brandi

**Affiliations:** 1 Bone Metabolic Diseases Unit, Department of Biomedical, Experimental and Clinical Sciences, University of Florence, Florence, Italy; 2 Department of Medical Sciences, Division of Endocrinology, Diabetes and Metabolism, University of Turin, Corso Dogliotti 14, 10126 Turin, Italy; 3 Endocrinology Unit, Fondazione IRCCS Ca’ Granda Ospedale Maggiore Policlinico, Department of Clinical Sciences and Community Health, University of Milan, Milan, Italy; 4 Department of Clinical and Experimental Medicine, University of Pisa, Pisa, Italy; 5 Department of Clinical Medicine and Surgery, Endocrinology Unit, University Medical School of Naples, Via Sergio Pansini 5, 80131 Naples, Italy; 6 Department of Information Engineering, University of Florence, Florence, Italy; 7 Azienda Ospedaliera Regionale San Carlo, Potenza, Italy; 8 Endocrinology Unit, Department of Internal Medicine and Medical Specialties (DiMI), University of Genoa, Genoa, Italy; 9 Department of Biomedical Sciences, Humanitas University, 20090 Pieve Emanuele, Italy; 10 Endocrinology, Diabetology and Andrology Unit, Humanitas Clinical and Research Center-IRCCS, 20089 Rozzano, Italy; 11 Unit of Endocrinology, Department of Medical Specialties, Azienda Ospedaliero-Universitaria Di Modena, Modena, Italy; 12 AUO-Careggi University Hospital, Florence, Italy; 13 Endocrinology Unit, Department of Medical Sciences and Public Health, University of Cagliari, Cagliari, Italy; 14 Department of Endocrinology and Diabetology, Azienda Ospedaliera San Giovanni Addolorata, Rome, Italy; 15 Endocrinology Unit, IRCCS Ospedale Policlinico San Martino, Genoa, Italy; 16 Fondazione Italiana Di Ricerca Sulle Malattie Dell’osso: F.I.R.M.O, Via San Gallo 123, 50100 Florence, Italy

**Keywords:** Chronic hypoparathyroidism, rhPTH (1–84), Natpar^®^, Treatment

## Abstract

**Purpose:**

The main purpose of this study was to investigate the effects of 12 months of rhPTH (1–84) (Natpar^®^) treatment in a cohort of patients selected according to the indications of hypoparathyroidism guidelines. The use of recombinant human PTH (1–84) [rhPTH (1–84)] is approved as hormonal replacement therapy in patients with hypoparathyroidism not adequately controlled with conventional therapy.

**Methods:**

It is a multicenter, observational, retro-prospective, open label study. Eleven Italian Endocrinological centers, members of Hypoparathyroidism Working Group of the Italian Society of Endocrinology (HypoparaNET) were involved. Main outcome measures were serum and urinary calcium and phosphate concentration, calcium-phosphate product, renal function, oral calcium and vitamin D doses, and clinical manifestations.

**Results:**

Fourteen adult subjects, affected by chronic hypoparathyroidism, were treated with rhPTH (1–84) for 12 months. At 12 months of rhPTH (1–84) treatment, 61.5% of patients discontinued calcium supplement and 69.2% calcitriol. Mean albumin-adjusted total serum calcium levels quickly normalized after initiation of rhPTH (1–84) treatment compared to baseline (*p* = 0.009), remaining in the normal range until 12 months. Rare hypo-hypercalcemia episodes were reported. Renal function was maintained normal and no renal complications were reported. Serum and urinary phosphate and urinary calcium were maintained in the normal range. Mean phosphatemia levels linearly decreased from 3 months up to 12 months compared to baseline (*p* = 0.014). No severe adverse events were described.

**Conclusions:**

Biochemical and clinical results confirm the efficacy and safety of rhPTH (1–84) therapy, which represents an important option for hypoparathyroid patients unresponsive to conventional therapy.

## Introduction

Hypoparathyroidism (HypoPT) is a rare endocrinological disease characterized by serum calcium or ionized calcium concentration below the normal range, accompanied by undetectable or inappropriately low levels of parathyroid hormone (PTH) [1]. The most common cause of HypoPT is neck surgery, followed by autoimmune, genetic and other causes [2].

The mainstay of conventional pharmacological management is supplemental oral calcium and vitamin D (calcitriol or analogs of active vitamin D) [2–4]. This pharmacological treatment usually controls serum calcium levels in HypoPT, however, it does not replace the missing hormone and sometimes high doses are often needed, raising long-term complications such as extra skeletal calcifications and renal function impairment [2–5].

In the first attempts to use parathyroid hormone peptides in HypoPT, Winer et al. showed that the synthetic PTH (1–34), biological active N-terminal peptide of PTH, could be used as an effective hormonal treatment in pediatric and adult patients [6]. The short half-life of PTH (1–34) required at least twice daily subcutaneous injections to control serum calcium over a 24 h period of time with smaller amounts of supplemental calcium and vitamin D, without effects on urinary calcium [7–10]. In Italy, teriparatide [rhPTH(1–34)] 20 µg/daily was approved for severe post-menopausal osteoporosis in 2004, and subsequently in 2013 the reimbursement of teriparatide with doses from 20 to 80 µg/daily was approved also for patients affected by severe chronic HypoPT not adequately controlled with conventional treatment. Teriparatide was initially approved with a limit of two years period treatment then extended to three years [11], and currently with no time limit, although recommending surveillance on the risk of osteosarcoma. In 2018, an Italian prospective open-label investigation, conducted on 42 adult patients with postsurgical HypoPT treated with teriparatide 40 µg/daily (20 µg/twice daily) for 24 months, confirmed Winer’s results [12–14]. However, no clinical trials for registration on teriparatide treatment have been conducted in patients with HypoPT, and no efficacy and safety data are available, especially considering the prolonged use of doses greater than 20 µg daily in young subjects and the potential risk of osteosarcoma [14].

The use of the recombinant human PTH (1–84) [rhPTH (1–84)], the native human hormone missing in HypoPT, ushered in the official therapeutic era of full hormone replacement therapy in patients not adequately controlled with conventional therapy [3, 4, 15–18]. It was approved in 2015 by FDA in the USA, with a “black box” warning related to the potential risk of osteosarcoma, but without time limit of use [19, 20]. Two years later, the European Medicines Agency (EMA) recommended granting a conditional marketing authorization in the European Union (EU) for Natpar^®^ [rhPTH (1–84)] as an “orphan medicine”. However, this treatment has been approved and reimbursed so far only in some Northern European countries, in Germany, and in Greece [21, 22]. In Italy, until May 2020 it was possible to prescribe the drug through the National Fund of Italian Drug Agency for the use of orphan drugs as treatment of rare diseases. This led to the prescription of the drug to a few tens patients who did not respond to either conventional therapy or therapy with teriparatide, had finished the 36-month period of teriparatide treatment. Unfortunately, after 2020, Natpar^®^ was classified in Italy as a medicine subject to limited medical prescription, not reimbursed by the National Health System [23]. This created considerable uncertainties and problems because patients treated with Natpar^®^ found themselves in a short time without reimbursed therapy. Two years ago, eleven Italian endocrinological specialized centers, members of Hypoparathyroidism Working Group [24], within the Italian Society of Endocrinology (SIE), conducted a retro-prospective observational study on HypoPT patients treated with Natpar^®^, selected according to the criteria of the available HypoPT guidelines [3, 4], to describe efficacy and safety of this treatment for at least 12 months. The results of this study are described below.

## Materials and methods

### Study population

The study included 14 adult subjects, affected by chronic HypoPT post-neck surgery or other causes, treated with rhPTH (1–84) (Natpar^®^) for 12 months (between January 2018 and December 2020) (Table 1). Eleven Italian Endocrinological centers participated in this study.

**Table 1:** General characteristics of the study group at baseline

Parameters	Frequency (%)
Sex female	13/14 (92.8)
Cause of HypoPT	
Post-surgical	11/14 (78.5)
Autoimmune polyendocrinopathy syndrome	2/14 (14.2)
Idiopatic	1/14 (7.1)

	Mean	SD	Range (min–max)
Age (years)	50.5	12.9	26–75
Body mass index (kg/m^2^)	28.7	7.5	20.2–42
Duration of Hypoparathyroidism (years)	25.3	16.5	3–56
Laboratory variables			
PTH (pmol/l;n.v.: 1.3–7.6)	0.5	0.3	< 0.3–1.0
Albumin-adjusted serum calcium (mmol/l;n.v.: 2.1–2.5)	1.92	0.16	1.62–2.12
Serum phosphate (mmol/l; n.v.: 0.8–1.58)	1.35	0.28	0.64–1.74
Urine calcium (mmol/24 h; n.v.: 2.5–7.5)	5.14	2.2	2.5–8.7
Serum creatinine (µmol/L; n.v.: 49–90)	70	7.9	53–86.6
25 oh vitamin D (ng/ml; n.v.: 30–100)	29.43	7.60	16–42
Serum magnesium (mmol/l; n.v.: 0.6–1)	0.7	0.1	0.7–0.94
Prescribed calcium:	Frequency (%)		
0–2000 mg/day	7/14 (50)		
> 2000 mg/day	7/14 (50)		
Prescribed calcitriol:	Frequency (%)		
≤ 0.25 μg/day	0/14 (0)		
0.25–0.5 μg/day	2/14 (14.2)		
≥ 0.5 μg/day	12/14 (85.7)		

### Inclusion and exclusion criteria

Patients enrolled for this observational study met the following inclusion criteria: diagnosis of chronic HypoPT by at least one year (the diagnosis of HypoPT was based on serum calcium and PTH levels below the lowest normal limits or inappropriate levels of PTH for calcemia); indication to treatment with rhPTH (1–84) according to the criteria of the HypoPT guidelines (3, 4) namely (1) inadequate control of the serum calcium concentrations despite conventional treatment; (2) need of high oral calcium/vitamin D doses (oral calcium > 2.5 g or > 1.5 µg of active vitamin D); (3) renal complications as hypercalciuria, renal stones, nephrocalcinosis, kidney stone, or reduced creatinine clearance or eGFR (< 60 mL/min); (4) hyperphosphatemia and/or calcium-phosphate product > 55 mg^2^/dl^2^ or 4.4 mmol/^2^l^2^; (5) gastrointestinal malabsorption; (6) reduced quality of life; or, in Italy, discontinuation of treatment with teriparatide either due to intolerance or due to end of reimbursement period of the drug.

Patients were excluded if they had other pathologies or drugs interfering with bone metabolism, or in case of contraindications as described in the technical data sheet [19].

### Study design

This is a 1-year, multicenter, observational, retro-prospective, open label study. Clinical, biochemical and pharmacological data were analyzed at the following times: baseline, after 2 weeks and then monthly up to 12 months. Enrolled patients self-administered a subcutaneous once daily injection of rhPTH (1–84). The management of rhPTH (1–84) dosages and the simultaneous reduction of calcitriol and calcium carbonate/citrate doses were managed and supervised by specialist doctors according to Natpar^®^ technical data sheet [22]. In particular, the initial treatment was with 25 or 50 µg once daily by subcutaneous injection in the thigh (alternating the thigh every day). Initially, in patients taking calcium supplements, the dose of the supplement usually unchanged and in patients who took an active form of vitamin D, the dose was reduced by 50% if serum calcium level was above 1.87 mmol/l (7.49 mg/dl). The pre-dose serum calcium concentration was measured within 2–5 days. The adjustment of the active form of vitamin D or the calcium supplement or both doses was based on serum calcium level and clinical evaluation, according to technical data sheet [22]. The dose of rhPTH (1–84) could be increased in increments of approximately 25 µg every 2–4 weeks, up to a maximum daily dose of 100 µg [22]. Serum calcium was measured 2–5 days after each supplementation therapy change. The main targets of rhPTH (1–84) treatment were based on the recent guidelines of chronic HypoPT (3, 4), in particular: (1) to prevent signs and symptoms of hypocalcemia; (2) to maintain the serum calcium concentration slightly below normal (ie, no more than 0.5 mg/dl below normal) or in the low normal range; (3) to maintain the calcium-phosphate product to below 55 mg^2^/dl^2^ or or 4.4 mmol/^2^l^2^; (4) to avoid hypercalciuria; (5) to avoid hypercalcemia; and (6) to avoid renal (nephrocalcinosis/nephrolithiasis) and other extra skeletal calcifications.

The main objective of this study was to describe the effects of 12 months rhPTH (1–84) treatment on biochemical indices and clinical manifestations in a cohort of adult subjects with chronic HypoPT, selected according to the criteria of recent HypoPT guidelines [3, 4], followed by Italian endocrinological centers.

### Assays

Serum calcium, phosphate, magnesium, and creatinine were measured by automated techniques. Serum calcium was adjusted for albumin by the following formula: 0.8 (4.0–patient’s albumin) + serum calcium [25]. Urinary calcium was measured by colorimetric method, urinary phosphate by potentiometric method, and 25 OH vitamin D by immunochemiluminescent methods. Glomerular Filtration Rate (eGFR) was calculated with Cockcroft-Gault equation.

### Ethics

All investigations were conducted in accordance with the Declaration of Helsinki. In accordance with the Italian Drug Agency instructions, all patients were required to sign an informed consent statement, allowing their anonymized information to be used for data analysis. Patient records were anonymized and deidentified before analysis. The study was approved by the Institutional Review Board (Comitato Etico Area Vasta Centro, AUOC, Florence, Italy) [number: 10641_oss; 16 May 2017]. Informed consent was collected in accordance with General Authorization to Process Personal Data for Scientific Research Purposes (Authorization no. 9/2013, The Italian Data Protection Authority).

### Statistical analyses

Analysis of frequencies and descriptive statistics were performed using the IBM Statistical Package for Social Sciences (SPSS 20.0) for Windows (IBM, Armonk, NY, USA). Data are presented as mean ± SD (Standard Deviation), unless otherwise stated. Repeated measures-related differences were evaluated by using Student’s *t* test for paired sample. For all the variables that did not meet the assumptions for parametric analysis, the Wilcoxon Signed-Rank Test was employed to assess paired data. A *P* value (*p*) of less than or equal to 0.05 was considered as statistically significant.

## Results

### Baseline characteristics

This study identified a cohort of 14 patients affected by chronic HypoPT, and baseline characteristics of study group are reported in Table 1. Most patients were women (92.8%; n:13), and the mean age was 49.5 years (SD 12.9). The main forms of HypoPT included post-surgical HypoPT (11/14), followed by 2 cases of autoimmune HypoPT (Autoimmune polyendocrinopathy syndrome type 1; APS-1), and 1 case of primary idiopathic HypoPT.

The reasons for switching to rhPTH (1–84) treatment in these patients were: frequent symptomatic episodes of hypocalcemia (*n*:10/14) despite standard therapy with calcitriol and calcium supplement, and gastrointestinal intolerance/malabsorption to supplementation calcium (*n*:3/14); or renal complications (kidney stones, nephrocalcinosis) due to long-term therapy with standard therapy (*n:*4/14). In particular, 8 patients showed difficulty in normalizing serum calcium levels despite conventional therapy, 2 patients, in addition to this, had also gastrointestinal intolerance/malabsorption to conventional therapy, 3 patients had renal complications and 1 had renal complications and gastrointestinal intolerance/malabsorption to conventional therapy. Eight out of 14 patients had previously been treated with teriparatide, administered subcutaneously with a mean dose of 20 μg twice a day (SD 10.6, range 20–60 μg/day; mean treatment period: 19.1 months, range 3 weeks–24 months). Among them, no patient stopped conventional therapy with calcium carbonate and calcitriol during teriparatide treatment. High doses of calcium (> 1.5 mg/day) and calcitriol (> 2.5 μg/day) were necessary in 3 patients despite ongoing teriparatide, in order to maintain normal calcium levels and control the clinical symptoms associated with hypocalcemia. Five out 8 patients described paresthesia/tingling/cramps due to hypocalcemia episodes despite adjunct therapy with teriparatide. Moreover, adverse events reported were: nausea (*n*: 1), headache (*n*: 2), muscle aches (*n*: 1); the symptoms disappeared when teriparatide was discontinued. Therefore, 3 out of 8 patients discontinued teriparatide treatment, one after 3 weeks and two after 18 months, while the other patients were treated with teriparatide up to 24 months. After discontinuing teriparatide treatment, patients were again treated with conventional therapy prior to switching to rhPTH (1–84).

At baseline visit, before starting rhPTH (1–84) treatment, the mean dose of oral calcium supplement was 2884.6 mg/day (SD 1792.9; range 1000–6000 mg/day), and the mean dose of calcitriol was 1.19 μg/day (SD 0.68; range 0.5–3 μg/day). Despite this standard treatment, baseline mean albumin-adjusted total serum calcium level was 1.92 mmol/l (SD 0.16, range 1.62–2.17; normal range values: 2.1–2.5 mmol/l). Only 3 patients had albumin-adjusted total serum calcium levels equal to 2–2.12 mmol/l [i.e. at the lower limit of the reference range or below 0.12 mmol/l (0.5 mg/dl) as recommended by the guidelines] [3, 4]. In addition, 2 patients had hyperphosphatemia and 4 hypercalciuria (urine calcium excretion > 4 mg/Kg/day) at baseline. Patients took an average of about 2000 IU (SD 1161, range 833–3333 UI day) of cholecalciferol per day.

### Changes in calcium and calcitriol doses and biochemical results during rhPTH (1–84) treatment

Thirteen subjects reached the 1-year time point and only 1 stopped treatment at 6 months, not for adverse events. Initial mean dosage of rhPTH (1–84) at baseline was 50 μg/day (*n*:12, 85.7%) and 25 μg/day (*n*:2, 14.2%). At 6 months, 35.7% of the patients took 50 μg/day rhPTH (1–84) (*n*:5), 35.7% 100 μg/day (*n*:5), followed by 75 (*n*:2) and 25 (*n*:2) μg/day. At 12 months, 7 patients (53.8%) took 100 μg/day rhPTH (1–84), 4 patients (30.7%) 75 μg/day, and 2 patients (15.3%) 50 μg/day. Table 2 shows changes over 12 months in mean doses of calcium, calcitriol and rhPTH (1–84). At 3 months of rhPTH (1–84), 42.8% of patients discontinued oral calcium supplementation, at 6 months 50%, and at 12 months 61.5%. Regarding calcitriol, at 3 months 50% discontinued the treatment, at 6 months 57.1%, and at 12 months 69.2%. The remainder took a mean dose of calcium equal to 423 ± 759.5 mg/day and calcitriol 0.21 ± 0.56 µg/day. A statistically significant decrease in the mean dose of calcium over the follow up was appreciated, starting from the third month compared to baseline (*χ*^2^_(4)_ = 35.50; *p* < 0.001). In particular, the post-hoc comparison using the Wilcoxon rank test showed a statistically difference at 3 months (*Z* = − 2.64; *p* = 0.008), 6 months (*Z* = − 2.94; *p* = 0.003), 9 months (− 2.84; *p* = 0.003), and 12 months compared to baseline (− 2.94; *p* = 0.003), compared to baseline. As regards the values of mean dose of calcitriol, a statistically significant decrease was observed starting from the third month (*χ*^2^_(4)_ = 40.46; *p* = 0.001). The post-hoc comparison showed a statistically difference between the baseline and 3 months (*Z* = − 2.68; *p* = 0.007) 6 months (*Z* = − 3.08; *p* = 0.002), 9 months (*Z* = − 3.08; *p* = 0.002), and 12 months (*Z* = − 3.08; *p* = 0.002).

**Table 2:** Changes over time mean doses of calcium, calcitriol supplements and rhPTH (1–84)

Medications	Baseline		3 months		6 months		12 months	
	Mean ± SD	Range	Mean ± SD	Range	Mean ± SD	Range	Mean ± SD	Range
Calcium carbonate, mg/day	2884 ± 1792.9	1000–6000	846.1 ± 965.7	0–4000	523.08 ± 740.7	0–2500	423.08 ± 759.5	0–2500
Calcitriol, µg/day	1.19 ± 0.68	0.50–3	0.36 ± 0.65	0–2	0.21 ± 0.56	0–2	0.21 ± 0.56	0–2
rhPTH (1–84), µg/day	46.4 ± 9.07	25–50	64.2 ± 25.4	25–100	67.8 ± 28.4	25–100	82.1 ± 20.6	50–100

**Fig. 1:**
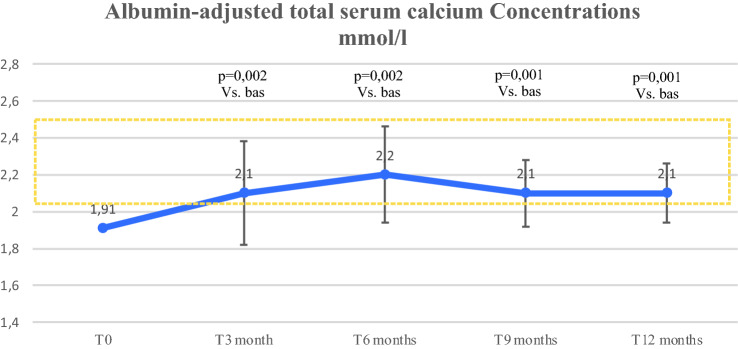
Changes over time in albumin-adjusted total serum calcium concentration. Circles are means, bars are SDs, and shaded area identifies the therapeutic target of calcium level. *T* time visit, *p* p value

Mean albumin-adjusted total serum calcium levels quickly normalized after initiation of rhPTH (1–84) (mean value after 2 weeks: 2.15 mmol/l, SD 0.3) compared to baseline (1.92 mmol/l, SD 0.16, Z = − 2.62, *p* = 0.009), with a statistically significant increase (*χ*^2^_(7)_ = 26.10; *p* = 0.001), remaining in the normal range until the end of the 12 months (Fig. 1). In the first 3 months of treatment with rhPTH (1–84), 8 episodes of hypocalcemia (5/14), and 1 episode of hypercalcemia (2.74 mmol/l) in 1 patient were described. No cases of hypercalcemia were observed in the following months. Only 1 patient tended to have albumin-adjusted total serum calcium values below the normal range. The latter patient was affected by post-surgical HypoPT from 3 years, not associated with any other known pathologies, except obesity grade 3 (BMI: 42). Although at 4 months, the patient reached the maximum available dose of rhPTH (1–84) 100 μg/day, the level of calcemia increased on average by only 0.12 mmol/l; therefore, the supplementation of calcium and calcitriol was maintained along with the hormone replacement therapy.

Mean urinary calcium excretion levels showed a significant increase after one month of treatment with rhPTH (1–84) compared to baseline (Z = − 2.76; *p* = 0.006), albeit remaining within the normal range and maintaining similar values over the follow-up.

**Fig. 2:**
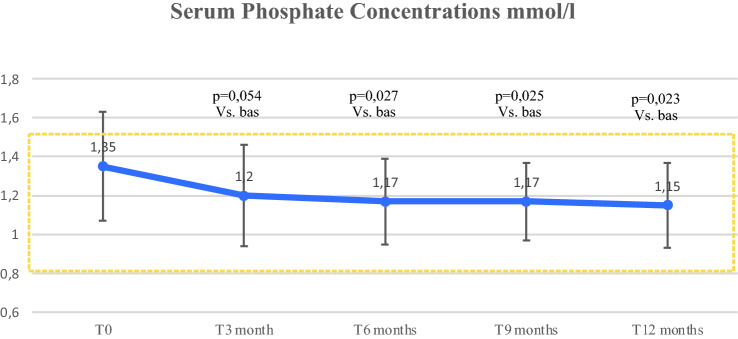
Changes over time in serum phosphate concentration. Circles are means, bars are SDs, and shaded area identifies the therapeutic target of serum phosphate level. *T* time visit, *p* p value

Mean phosphatemia level linearly decreased from 3 months until 12 months compared to baseline (*χ*^2^_(4)_ = 12.48; *p* = 0.014), maintaining the levels within the normal range (Fig. 2). Four out of 14 patients (28.5%) had hyperphosphatemia at baseline and no episodes were described in the following months.

Mean phosphaturia levels showed values within the normal range for all 12 months without statistically significant differences.

Calcium-phosphate product (Ca*P) was maintained < or 4.4 mmol/^2^l^2^ (55 mg^2^/dl^2^) during treatment with rhPTH (1–84) and over the follow-up period (mean value at baseline: 2.60 mmol^2^/l^2^, SD 0.59; at 6 months: 2.4, SD 0.4; at 12 months: 2.3, SD 0.4). A statistically significant reduction in Ca*P was observed between baseline assessment and 12 months (*F*_(4,48)_ = 2.96; *p* = 0.03).

Mean serum creatinine, estimated Glomerular Filtration Rate (eGFR) and 25 OH vitamin D levels remained within the normal range (mean value 33.1 ng/ml, SD 6.1, range 18–51 ng/ml) both at baseline and during follow up period with rhPTH (1–84) treatment, without statistically significant differences. The mean daily cholecalciferol dosage taken was equal to 2572 UI/day (SD 1523, range 1000–6666 UI day) during rhPTH (1–84) treatment.

### Clinical manifestations and adverse events during rhPTH (1–84) treatment

No serious adverse events were described in 12 months of treatment with rhPTH (1–84). One patient had an episode of diarrhea after the first administration of rhPTH (1–84), which resolved after 2 days without rhPTH (1–84) discontinuation. During the rhPTH (1–84) treatment period, no new clinical manifestations such as kidney stones or nephrocalcinosis were described with renal ultrasound, nor extra skeletal calcifications, cataracts, fragility bone fractures and cardiovascular complications were reported. Regarding neuromuscular symptoms, at baseline 5 out of 14 patients had muscle cramps/paresthesia/tingling, which regressed during treatment with rhPTH (1–84) within the first month, except 1 patient whose symptoms lasted for the first 6 months. No changes in renal function were reported during treatment with rhPTH (1–84), with maintenance of serum creatinine levels within the normal range.

## Discussion

This observational study provides insight into benefits and safety of rhPTH (1–84) treatment in HypoPT patients, selected according to the criteria of the available HypoPT guidelines [3, 4]. The number of evaluated patients is limited, however it must be considered the rarity of the pathology and difficulties in prescribing rhPTH (1–84) in Italy. For the same reasons, a retro-prospective study was conducted in order to collect the greatest number of data, despite the intrinsic limitations of a retrospective evaluation. This study confirmed the efficacy of 1-year rhPTH (1–84) treatment in adult patients not adequately controlled with conventional therapy (calcium and active vitamin D or analogs), and showed an improved response to rhPTH (1–84) compared to teriparatide in the 8 patients previously treated with this drug. However, in Italy the prescription or continuation of rhPTH (1–84) treatment has recently been limited by the transition to drug not reimbursable by the public Italian National Health System.

Patients with HypoPT are usually treated with standard therapy; however, in some cases, pharmacological management can become difficult and challenging even for expert endocrinologists [3, 4, 18, 24, 26]. Conventional treatment, especially in case of need for high doses of calcium and calcitriol supplements, may cause long-term complications such as hypercalciuria, renal stones, nephrocalcinosis, other ectopic calcifications, and impaired renal function [2–5, 14, 17, 18, 27–29]. Moreover, compliance with standard therapy is often poor and this treatment is not able to improve quality of life usually impaired in these patients [30–33]. In our cohort of patients, the main indications for switching to hormone replacement therapy were: inadequate control of albumin-adjusted total serum calcium level despite conventional treatment, kidney stones, and/or gastrointestinal intolerance to supplementation calcium/malabsorption [3, 4, 18]. In more than half of the cases, the patients had already been treated with teriparatide (average dosage: 20 μg twice/day), however this therapy has an important limitation for this chronic pathology: the absence of therapeutic efficacy for dosages equal to 20 μg/day, the only dosage for which safety data are available from randomized and controlled studies in osteoporotic patients [4, 12–14]. Furthermore, even at dosages above 20 mcg/day, therapeutic efficacy in biochemical and clinical terms is not always achieved as described in our patients.

In our study, the initial rhPTH (1–84) treatment varied from 25 to 50 μg/day, with a subsequent tailoring of therapy in the first 3 months, up to a maximum dosage of 100 μg/day. At 12 months, more than half of the patients (53.8%) were taking 100 μg/day and the discontinuation of calcium and calcitriol supplementation was high (61.5 and 69.2% respectively), with the maintenance of normal albumin-adjusted total serum calcium. A reduction in oral calcium and calcitriol supplementations was appreciated as early as at the first month, but was statistically significant starting from the third month, whilst maintenance of adequate levels of albumin-adjusted total serum calcium. These results confirm what was described in the REPLACE randomized, double-blinded, placebo-controlled phase 3 clinical trial (24 weeks), including 90 subjects with HypoPT compared to placebo (*n* = 44) and in its subsequent open-label extensions called “REPEAT” (32 patients; 24 weeks) and “RACE” (49 patients; 5 years) [15, 34, 35]. In the REPLACE study, subjects were initially treated with rhPTH (1–84) at a dose of 50 µg daily, titrated as needed to 75 or 100 µg, and most subjects (52%) needed the highest dose. Over half of the study patients reduced calcium supplements and active vitamin D by 50%, along with maintenance of the serum calcium at 6 months, as compared to virtually none in the placebo group (53 vs. 2%; *p* < 0.001) [15]. Moreover, a marked difference was also documented in the percentage of patients who could eliminate active vitamin D, while taking no more than 500 mg of oral daily calcium (43 vs. 5%; *p* < 0.001) [15]. In the REPEAT study, at the end of the following 6 months, 58% (14/24) patients eliminated both oral calcium and calcitriol [34]. Subsequently, the RACE study and other USA, prospective open-label trials conducted on small groups of adult patients with HypoPT, showed significant reductions in calcium and calcitriol supplementation after 1 year of rhPTH treatment (1–84) and progressive reduction of calcium and active vitamin D requirements over 8 years [35–38].In our study, mean albumin-adjusted total serum calcium levels quickly normalized after initiation of rhPTH (1–84) treatment already after 2 weeks compared to baseline, remaining in the normal range until the end of the 12 months period of observation, with a statistically significant increase compared to baseline. Previous investigations like REPLACE and RACE studies also described a rapid and significant increase in serum calcium levels within 2–4 weeks of the initiation with rhPTH treatment (1–84), and subsequent prospective open-label studies demonstrated the efficacy of rhPTH (1–84) to control serum calcium levels in both short- and long term until 8 years [15, 35–39]. Hypercalcemia and hypocalcemia episodes usually result uncommon [15, 35–39]. In the present study, rare albumin-adjusted total serum calcium oscillations were described mainly in the first 3 months, with a subsequent normalization. Certainly, the first months of rhPTH (1–84) treatment involve rhPTH titration (1–84) and adaptation to the new treatment; therefore, close monitoring of biochemical parameters and related clinical manifestations is necessary [3, 4], and patients should be well educated to recognize neuromuscular symptoms of hypocalcemia. In our study, only one patient affected by post-surgical HypoPT, showed low mean levels of albumin-adjusted total serum calcium despite treatment with rhPTH (1–84). Unlike other enrolled patients, he had obesity grade 3. It can be hypothesized that patients with severe obesity may not respond adequately to rhPTH (1–84) therapy, even at the maximum doses. However, no specific data on this issue are reported in the literature, and according to the drug's technical data sheet, no dose adjustments are required based on body weight [22]. Published data suggest that patients with high body mass require higher doses of calcitriol probably due to accumulation of 1,25(OH)_2_D_3_ (perhaps in esterified form) in the adipose tissue or for an increased expression of the vitamin D receptor in this tissue [40]. Large-scale studies are needed to assess the response to treatments in patients with HypoPT, obesity and metabolic syndrome. Our study and other previously published studies showed no differences in response to rhPTH treatment (1–84) in terms of controlling albumin-adjusted total serum calcium levels between patients with post-surgical HypoPT and other forms [15, 35–39].

The control of urinary calcium levels is extremely important in these patients [2, 5, 27–29], and our study shows that, despite a tendency to an initial increase in mean urinary calcium excretion, this was maintained within the range for all 12 months of treatment. Previous investigations with rhPTH (1–84) described an overall reduction of 24 h urinary calcium levels after the first 6 months, with subsequent progressive reduction of urinary calcium excretion up to 5 years in the RACE study and up to 8 years in a small open label observational study [15, 35, 37].

Regarding the renal function, our study showed that serum creatinine and eGFR levels remained stable for all the first 12 months. The REPLACE and REPEAT studies did not describe the effect on creatinine and eGFR levels in the first 12 months of rhPTH (1–84) treatment. Subsequent prospective studies documented the maintenance of stable creatinine and eGFR levels after 1 year of treatment and up to 8 years [34, 35, 37]. Moreover, in our study, ultrasound renal monitoring analyzed showed no new onset of kidney stones. In previously published studies, only 1 prospective open-label study reported one episodes of nephrolithiasis (1/27 patients) in the fourth year of treatment with rhPTH (1–84) [36]. Other studies did not report kidney stones or nephrocalcinosis during treatment with rhPTH (1–84), although it was not the main objective of the investigation [38, 39].

As to the control of mean serum and urinary phosphate levels in response to rhPTH treatment (1–84), our study showed a significant decrease in phosphatemia from 3 months of treatment up to 1 year and stable levels of phosphaturia within the normal range. The REPLACE study and subsequent studies described a significant reduction in serum phosphate levels starting from 6 months compared to baseline, and the study with 5 years follow up described serum phosphorous levels uniformly lower than baseline throughout the period monitored [15, 41].

Calcium-phosphate product was maintained < or 4.4 mmol/^2^L^2^ (55 mg^2^/dL^2^) during treatment with rhPTH (1–84) and over the follow-up period with a statistically significant reduction at 12 months compared to baseline, as described in REPLACE and RACE studies, with maintenance of lower levels compared to baseline up to 5 years [14, 35, 41]. This result together with the control of urinary calcium should potentially reduce the long-term risk of extra-skeletal calcifications, however, this effect will need to be confirmed by ongoing studies.

In our cohort of patients, mean levels of 25 OH vitamin D were maintained above the target recommended by guideline (75 nmol/L or 30 ng/ml or higher) during treatment with rhPTH (1–84) [3], thanks to cholecalciferol supplementation. The rationale for using also this form of vitamin D in hypoparathyroid patients is both the utility in case of residual secretion of PTH and that it may be of importance to a number of cellular processes, may undergo hydroxylation to 1,25(OH)_2_D_3_ catalyzed by local hydroxylases in different tissues, and may have beneficial non-skeletal effects [3, 4, 18].

No significant differences were described between patients with post-surgical or autoimmune / idiopathic HypoPT in terms of response to rhPTH therapy (1–84). Lastly, regarding the safety of rhPTH (1–84), our study did not show serious adverse events as previously described by previous studies [15, 35–41].

## Conclusions

In conclusion, this study describes an Italian experience of eleven Endocrinological Centers that have followed patients with HypoPT treated with rhPTH (1–84), according to the criteria of the available HypoPT guidelines [3, 4], from 2018 to 2020. Our biochemical and clinical results confirm the efficacy of this treatment and add results on renal function and renal ultrasound monitoring in the first 12 months of treatment with rhPTH (1–84), confirming the safety of this treatment. In our study, clinical cases of difficult therapeutic management were selected, and although they are the minority of patients affected by HypoPT, they represent a great challenge for all endocrinologists who care for these patients. This study, in addition to describing the effects of treatment with rhPTH (1–84), underlines the difficulties of therapeutic management with conventional therapy, the issues related to treatment with teriparatide both in terms of its efficacy and lack of data on safety issues, and the current problems to access to treatment with rhPTH (1–84) for patients with HypoPT in Italy. Data on the long-term effects of rhPTH therapy (1–84) on various aspects such as skeletal dynamics, renal function, quality of life and complications are still missing. However, to date all the studies presented show good results in term of efficacy and safety until 8 years and a long-term monitoring study is currently underway [42]. We hope that therapeutic management of patients suffering from chronic HypoPT unresponsive to conventional therapy can improve in the near future also in Italy.

## Data Availability

All data generated or analyzed during this study are included in this published article.
